# The human physiological impact of global deoxygenation

**DOI:** 10.1007/s12576-016-0501-0

**Published:** 2016-11-15

**Authors:** Daniel Martin, Helen McKenna, Valerie Livina

**Affiliations:** 1 UCLH NIHR Biomedical Research Centre, Institute of Sport and Exercise Health, University College London Centre for Altitude Space and Extreme Environment Medicine, First Floor, 170 Tottenham Court Road, London, W1T 7HA UK; 2 Intensive Care Unit, Royal Free Hospital, Pond Street, London, NW3 2QG UK; 3 Royal Free Hospital, University College London Division of Surgery and Interventional Science, Pond Street, London, NW3 2QG UK; 4 National Physical Laboratory, Hampton Road, Teddington, TW11 0LW UK

**Keywords:** Oxygen, Hypoxia, Acclimatization, Physiological adaptation

## Abstract

There has been a clear decline in the volume of oxygen in Earth’s atmosphere over the past 20 years. Although the magnitude of this decrease appears small compared to the amount of oxygen in the atmosphere, it is difficult to predict how this process may evolve, due to the brevity of the collected records. A recently proposed model predicts a non-linear decay, which would result in an increasingly rapid fall-off in atmospheric oxygen concentration, with potentially devastating consequences for human health. We discuss the impact that global deoxygenation, over hundreds of generations, might have on human physiology. Exploring the changes between different native high-altitude populations provides a paradigm of how humans might tolerate worsening hypoxia over time. Using this model of atmospheric change, we predict that humans may continue to survive in an unprotected atmosphere for ~3600 years. Accordingly, without dramatic changes to the way in which we interact with our planet, humans may lose their dominance on Earth during the next few millennia.

## Introduction

Human dominion over planet Earth is driving profound changes that may culminate in extinction. Loss of natural vegetation and the burning of fossil fuels are altering our atmosphere at an alarming rate [1]. Two interconnected themes have received the most attention: the accelerated rise in atmospheric carbon dioxide concentration and the escalation of global temperatures. These changes are accompanied by natural phenomena with potentially catastrophic consequences, such as increasingly unpredictable climate subsystems and rising sea levels from polar ice cap recession [2–4]. If such environmental hazards were not a sufficient threat to the survival of Earth’s 7 billion plus human inhabitants, there is yet another concerning change already underway, global deoxygenation. Although the current volume of oxygen in our atmosphere is vast, it is diminishing inexorably, and yet this does not appear to be a priority for environmental concern. While the dynamics of oxygen decline is highly contentious, a new model of its nonlinear nature predicts total oxygen depletion within several thousand years [5]. This disruption of Earth’s fragile ecosystem could be the final straw for humans and the many other forms of life that rely on oxygen to generate energy. Here we discuss the biological significance of atmospheric oxygen, the proposed model of its decline, and its potential impact on human survival.

## Oxygen and Earth’s atmosphere

**Fig. 1:**
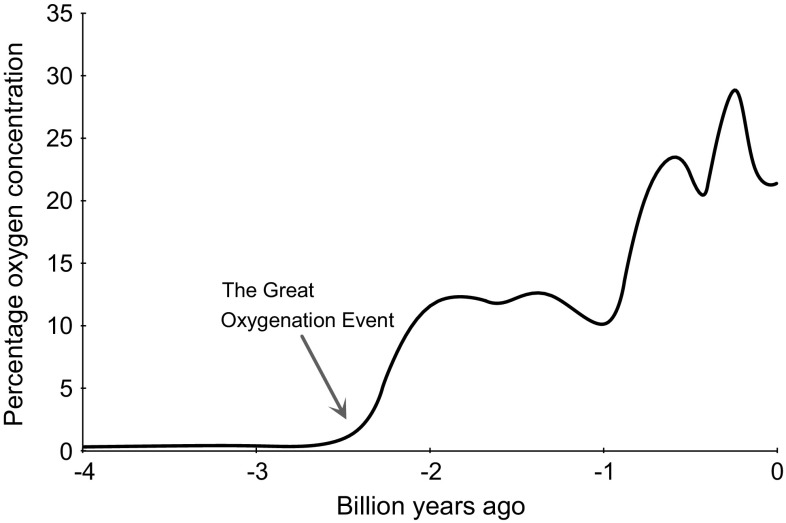
Approximate change in the concentration of oxygen in Earth’s atmosphere over the last 4 billion years [7]

Molecular oxygen (O_2_) is arguably one of the most important elements on Earth, particularly for the aerobic organisms that depend on it to release energy from carbon-based macromolecules. The concentration of oxygen in the atmosphere is 20.95% (209,460 ppm) but this has fluctuated markedly throughout history (Fig. 1). Earth is ~4.5 billion years old, and its early atmosphere contained virtually no oxygen whatsoever. The primitive atmosphere was instead formed mainly of hydrogen, with traces of methane and ammonia. Volcanoes then leached nitrogen and carbon dioxide into this mixture but it was not until the ‘great oxygenation event,’ ~2.3 billion years ago, that oxygen was released into the atmosphere by cyanobacteria that used carbon dioxide and sunlight to generate energy via photosynthesis [6, 7]. As photosynthesizing cells spread across the planet, they eventually developed into complex multi-cellular complex plants. This crucial event in the developing ecosystem changed the world from a barely habitable rock into the luscious green environment that we recognize today. Over time, oxygen became the predominant mechanism for energy to be generated by non-photosynthesizing cells, through oxidative phosphorylation in mitochondria. As billions of years passed by, an equilibrium was established whereby the concentration of oxygen fluctuated within a habitable range, between about 15 and 35%, which has been maintained from the beginning of the Cambrian period 540 million years ago until the present day (Fig. 1). Each surge in the atmosphere’s oxygen concentration was accompanied by a new burst of life, while the troughs were associated with downscaling and extinction of species. The oscillation of the atmospheric oxygen concentration around a level that optimally promotes the development of multicellular organisms is termed the global oxygen cycle [8]—but what are the margins for the existence of complex life on Earth, particularly humans? As global deoxygenation is a reality, understanding the impact of declining atmospheric oxygen on human physiology is of increasing importance. Defining the lower limit of atmospheric oxygen compatible with human survival will inform predictions of how long our species can persist on a planet undergoing progressive asphyxiation.

## The decline in global oxygen concentration

**Fig. 2:**
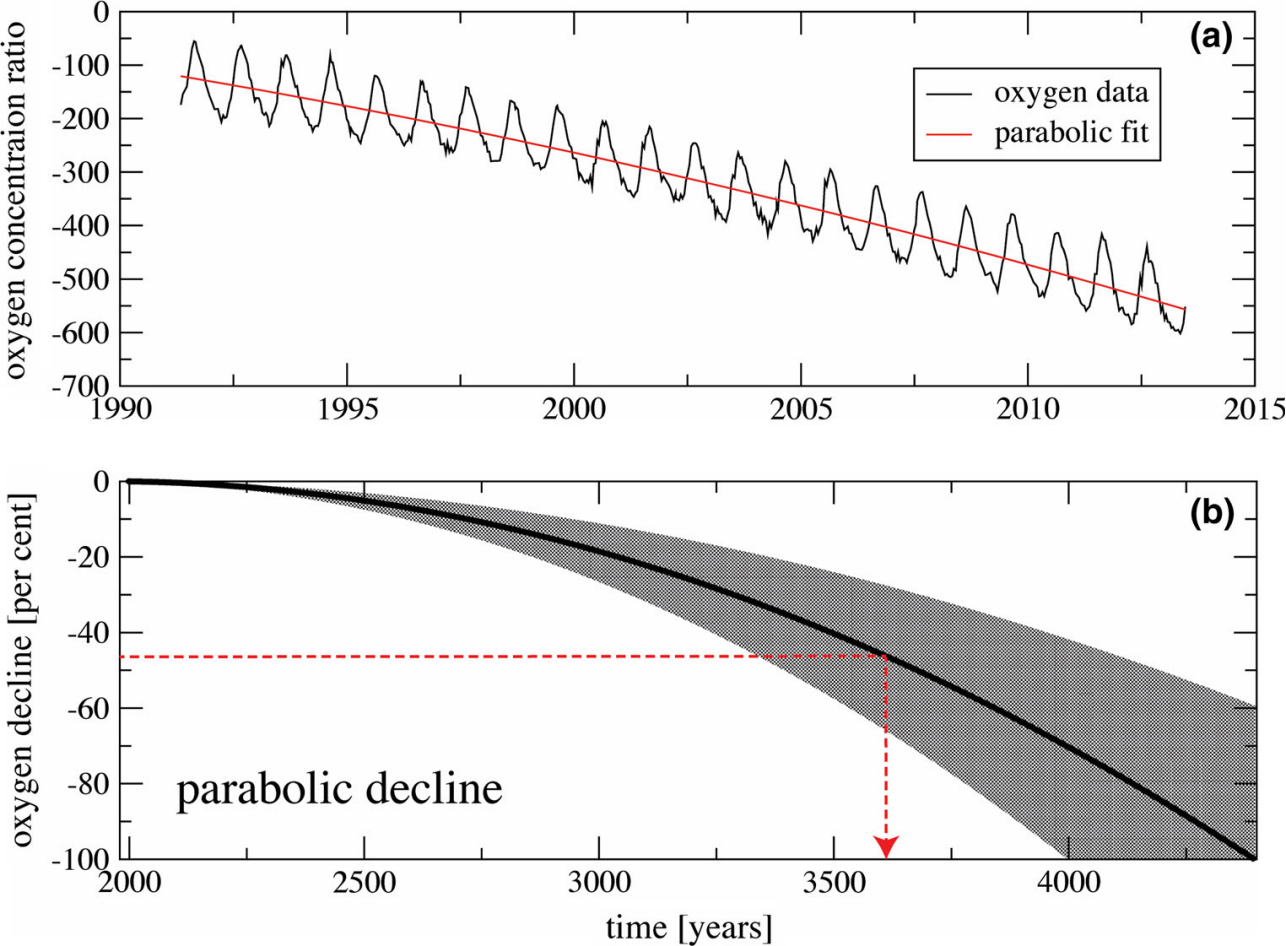
**a** The change in oxygen concentration ration over time recorded at ‘Alert Station’ in Canada, with a parabolic fitted decay curve [9]. **b** The projected decline based in oxygen concentration over time, using data from nine recording station in the Scripps Programme [9]. The uncertainty of the projection, based on the nine observational records around the globe, is shown in the* grey shaded area*. The* red dotted line* denotes lowest oxygen concentration likely to be tolerated by humans and time from now at which this will occur. Both figures reproduced with permission from [9]

Since 1989, detailed recordings from atmospheric air have been taken in order to monitor global concentrations of carbon dioxide and oxygen. Observational records of atmospheric oxygen concentration from several stations around the globe are maintained within the Scripps Institute Oxygen Programme (http://scrippso2.ucsd.edu/). Oxygen measurements are reported as changes in the oxygen/nitrogen (O_2_/N_2_) ratio of sampled air relative to a reference (air pumped in the mid-1980s and stored in the Scripps laboratory). The unit for these measurements is “per meg”, which means that a decline of 1 per meg is equivalent to a 0.0001% decline of oxygen concentration. Put another way, 1 per meg indicates one molecule out of 1,000,000 oxygen molecules, or roughly one molecule in 4.8 million molecules of air. Because natural variation of nitrogen concentration is much smaller than that for oxygen and its concentration is much higher, changes in O_2_/N_2_ ratio primarily reflect changes in oxygen concentration [9]. According to the Scripps Institute, the oxygen concentration is currently declining by ~19 per meg per year, equivalent to 4 ppm per year (Fig. 2).

From the original Scripps Institute data, it was hypothesized that the decline in atmospheric oxygen concentration was linear, however, as the dataset grows, it has been proposed that this may not be the case. One possible scenario is a parabolic decline [5]. Because the observed records span only a few decades, projection of this decay is highly uncertain and the complexity of the biogeochemical interactions makes such a projection a challenging task. However, this new mathematical model proposes that the horizon of oxygen decline will be reached much sooner than previously estimated from a linear model, leaving only a few thousand years until total oxygen depletion [5]. If we apply this parabolic projection to the currently available observed data, it predicts that the concentration of oxygen in the atmosphere will reach zero in ~4400 years, passing beyond the halfway point in ~3600 years (Fig. 2).

The dynamics of the oxygen decline is approximated by using a stochastic model:

$$Z\left( t \right) = T\left( t \right) + A\left( t \right)\cos \left( {2\pi \varphi \left( t \right)} \right) + \tau \left( t \right)\varPhi \left( t \right),$$

Here, trend *T*(*t*) is a real-valued function, *Φ*(*t*) is a stationary generalized random process, and *τ*(*t*) is a positive real-valued smooth function used to model the heteroscedasticity of the error term. As an example, *Φ*(*t*) can be continuous autoregressive moving average random process of order (*p*, *q*). The model realistically reproduces oxygen data as a combination of a declining global trend, seasonal periodicity, and smaller-scale fluctuations of higher frequency. This oscillating decline can be clearly seen in the Scripps Institute data (Fig. 2) and hypotheses to explain it have been previously been presented [10].

## Why is atmospheric oxygen concentration declining?

**Fig. 3:**
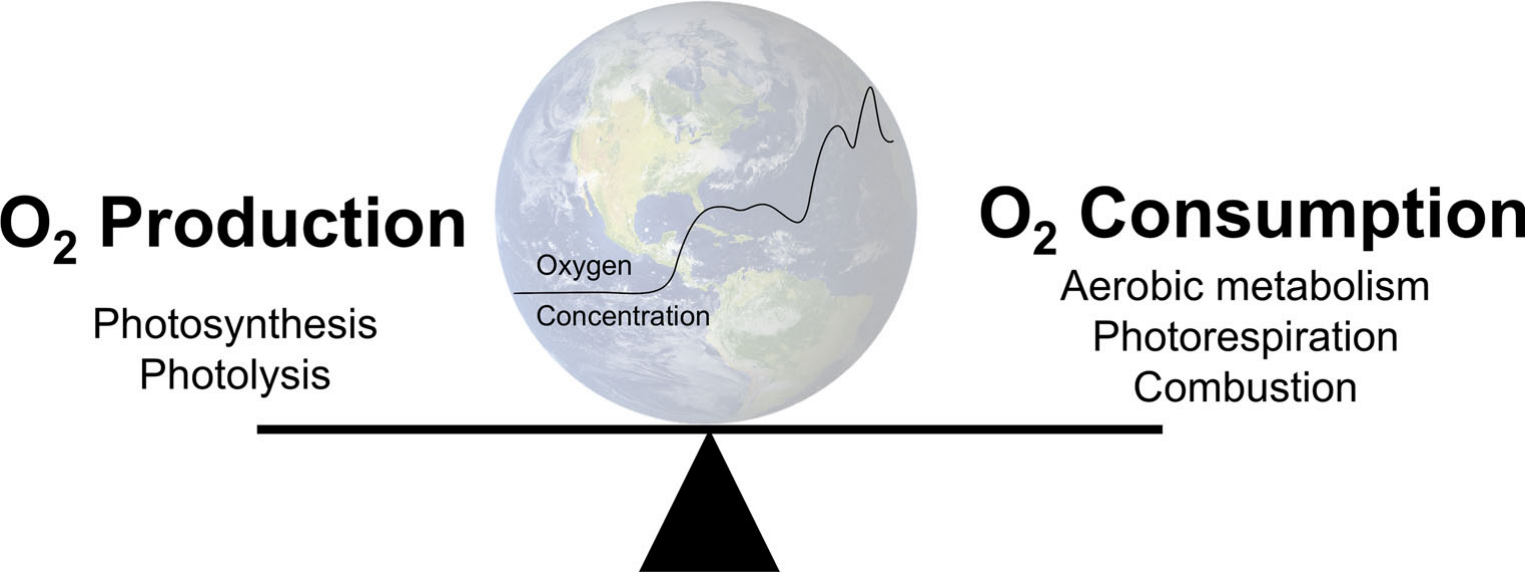
Processes that influence the global oxygen cycle and therefore the atmospheric concentration of oxygen

Earth’s atmosphere, like most biological systems, is in a constant state of flux governed by oxygen’s generation and destruction; natural processes that surround us every day (Fig. 3). The primary source of oxygen on Earth is photosynthesis, the generation of carbohydrates from carbon dioxide and water using sunlight as a catalyst (Eq. 1). Higher plants, algae, cyanobacteria, and prochlorophytes are all capable of photosynthesis. A tiny amount of molecular oxygen is also produced by the photolysis of water vapor in the upper atmosphere (Eq. 2). The end-users of molecular oxygen are aerobic life forms, including humans, which use it to generate the majority of their energy requirements (Eq. 3). Oxygen is also used during photorespiration, in which organic substrates are oxidized to yield ATP for energetic processes, in some photosynthesizing cells. The atmosphere and sea act as the main reservoirs for oxygen and major events such as natural fires can alter the balance to a minor degree. Therefore, in simple terms, if the number of plants decreases, the oxygen-generating capacity of the Earth is reduced; and if the number of animals (including humans) increases, then oxygen consumption will rise. Combustion of fossil fuels has a major impact on oxygen and carbon dioxide levels in the atmosphere, there being a correlation between fossil fuel-related global warming and depletion of oxygen from the oceans [11]. Furthermore, oxygen is consumed not only when fossil fuels are used in combustion, so the relation between decline of oxygen and rise in carbon dioxide is not linear. For example, oxygen is consumed in many oxidation processes in industry for materials manufacturing, where there is no direct combustion of fossil fuels. One of the uncertain factors in the model of oxygen decline lies in the rise of new technologies, which may appear “green” in terms of carbon dioxide emissions but at the same time would deplete oxygen—these need to be monitored in the context of the atmospheric oxygen decline. Waning of atmospheric oxygen concentration will also have knock-on effects in the oceans. Henry’s law determines the amount of gas that dissolves into a liquid and as the atmospheric partial pressure of oxygen (PatO_2_) declines, oxygen will diffuse out of the water leading to further oceanic deoxygenation, depriving the underwater world of the oxygen it requires to survive. It has been estimated that ~70% of atmospheric oxygen is produced in the oceans by photosynthesis in phytoplankton [12]. There is concern that uncontrolled global warming could lead to a catastrophic loss of this vital source of atmospheric oxygen through inhibition of photosynthesis [13].

Photosynthesis:

1 $$6 {\text{CO}}_{ 2} {\text{ + 6H}}_{ 2} {\text{O}} \to 6 {\text{O}}_{ 2} {\text{ + C}}_{ 6} {\text{H}}_{ 1 2} {\text{O}}_{ 6} .$$

Photolysis:

2 $$2 {\text{H}}_{ 2} {\text{O}} \to 4 {\text{H}}^{ + } {\text{ + e}}^{ - } {\text{ + O}}_{ 2} .$$

Aerobic metabolism:

3 $${\text{C}}_{ 6} {\text{H}}_{ 1 2} {\text{O}}_{ 6} {\text{ + 6O}}_{ 2} \to 6 {\text{CO}}_{ 2} {\text{ + 6H}}_{ 2} {\text{O}} .$$

## Biological impact of atmospheric deoxygenation

The biological effect of a gas is determined by its partial pressure, which, according to Dalton’s law, is equal to the product of barometric pressure and the fractional concentration of the gas in the mixture (Eq. 4). For example, at sea level, PatO_2_ is ~21 kPa (sea level barometric pressure 101 kPa × fractional concentration of oxygen 0.21). Thus, atmospheric hypoxia may result from either a decline in oxygen concentration (normobaric hypoxia) or a reduction in barometric pressure (hypobaric hypoxia). Differences in the biological responses to these situations are subtle but not completely insignificant [14]. For humans, the principal consequence of a fall in PatO_2_ is hypoxemia (a lack of oxygen in the blood), resulting in reduced delivery of oxygen to the tissues (tissue hypoxia). This occurs during ascent to high altitude, due to the exponential decline in barometric pressure. Above ~1500–2500 m, depending upon the individual, hypoxemia can lead to altitude-related illnesses, such as acute mountain sickness (AMS). Hypoxemia and tissue hypoxia can also result from many pathophysiological states that impair oxygen transport, such as respiratory and cardiovascular diseases. Humans have the ability to adapt to hypoxemia, through a process known as acclimatization, but the extent to which adaptation can compensate for the oxygen deficit depends on the magnitude of the deficit, and the time over which it occurs.

4 $${\text{Partial pressure of gas A}} = {\text{barometric pressure }} \times {\text{ fractional concentration of gas A}} .$$

## Adaptation to acute hypoxia

No clear definitions exist to define time-related exposures to hypoxia, but attempts have been made to unify the language used [15]. A significant and abrupt fall in PatO_2_ cannot be tolerated for more than a few minutes before cerebral hypoxia results in unconsciousness. Descriptions of death following sudden oxygen deprivation were common amongst early high-altitude aviators during World War II [16]. The “time for useful consciousness” on sudden exposure to a simulated altitude of 7620 m above sea level (PatO_2_ ~8 kPa) is ~4–5 min) [17]. The rapidity of the fall in PatO_2_ prevents any meaningful adaptation beyond hyperventilation and tachycardia, a desperate attempt to increase circulating blood oxygen levels. This almost immediate physiological change is brought about through oxygen sensing in the carotid bodies and their subsequent effect on the respiratory and cardiovascular centers of the brain.

## Adaptation to subacute hypoxia

**Fig. 4:**
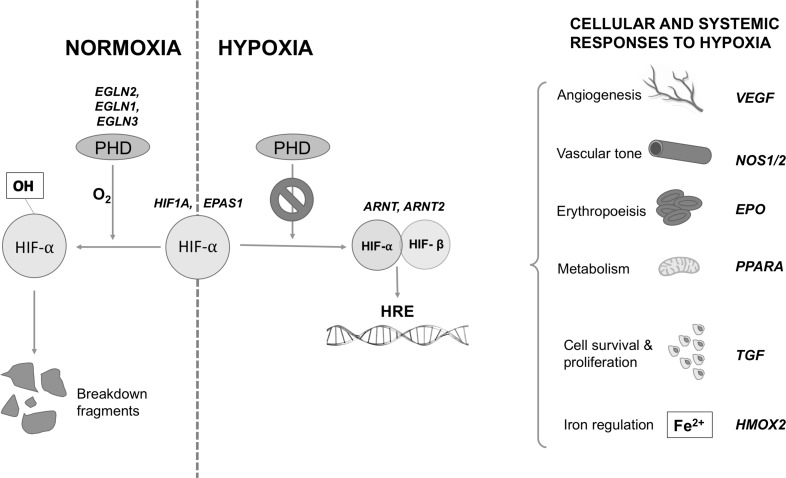
Examples of genes (in *red*) within the HIF pathway in which positive selection has been identified in high-altitude populations. Activation of the HIF response involves prolyl hydroxylases (PHD), which, in the presence of oxygen, hydroxylates HIFα thus targeting it for destruction by the ubiquitin–proteasome pathway. Under hypoxic conditions, HIFα persists to combine with the constitutively present HIFβ, and this dimer acts as a transcription factor, influencing the expression of over 100 genes, which possess hypoxia response elements in their promoter regions, and play a role in the cellular and systemic response to hypoxia. The HIF response involves increasing oxygen delivery to hypoxic tissues (through effects on angiogenesis, vascular tone, and erythropoiesis) as well as modifying cell metabolism, proliferation, and survival pathways. High-altitude positive selection has been demonstrated in all parts of this pathway, but none of the alleles affected have been demonstrated in more than one population. Data summarized from [31]. *HIF* hypoxia-inducible factor, *HRE* hypoxia response elements, *PHD* prolyl hydroxylase

With a more gradual exposure to hypoxia, as might be experienced by during a trek to high altitude, other biological systems have a chance to contribute through a process known as acclimatization. In addition to augmented ventilatory and cardiovascular responses, hypoxia is sensed at a cellular level, triggering the increased concentration of a gene regulator known as hypoxia inducible factor (HIF). The HIF complex up-regulates a variety of specific genes, all pertinent to the survival of a sustained hypoxic exposure (Fig. 4). This oxygen-sensing system is one of the oldest and most robust cellular regulators and is preserved throughout almost all life on Earth, signifying the importance of the ability to rapidly respond to changes in oxygen availability [18]. One important role of HIF is to increase the number of circulating red blood cells through the up-regulation of erythropoietin. This increases the oxygen-carrying capacity of the blood over a period of days to weeks and forms the backbone of short-term hypoxic adaptation. It is through this process that the summit of Mount Everest (8848 m), where PatO_2_ plummets to 7 kPa, can be reached without the use of supplemental oxygen [19].

## Lifelong hypoxic exposure

Chronic disease or long-term residence at high altitude can expose humans to a lifetime of hypoxemia. ~140 million people live permanently at high altitude (conventionally defined as >2500 m: the elevation that most people demonstrate a drop in the oxygen saturation of hemoglobin, SpO_2_) [20]; and whilst this is compatible with life, it is not without consequences for many. With increasing altitude of residence, chronic mountain sickness (CMS) and intrauterine growth retardation (IUGR) become more prevalent and an acceleration of pre-existing chronic respiratory diseases is observed [21]. Global atmospheric deoxygenation would lead to such complications being encountered at progressively lower elevations, and we would expect their incidence and severity increase on a global scale. CMS is characterized by excessive polycythemia, progressing to pulmonary hypertension, right ventricular failure, and death [22]. There is limited information about the frequency of and mortality from this disease in the present day as it is not a recognized classification in death certificates [21], but it will undoubtedly generate a significant and progressive disease burden as deoxygenation continues. The impact on human reproduction may have even more grave ramifications for population expansion and health. Fetal hypoxia, due to reduced maternal oxygenation and uteroplacental blood flow, reduces birth weight by an average of 100 g for every 1000 m above sea level [20, 23]. IUGR has wide-ranging and severe consequences throughout life. Low birth weights are linked to higher mortality in infancy, childhood, and later in life. Late (adult) morbidity and mortality may be due to the heightened risk of systemic hypertension, coronary heart disease, and diabetes observed in low-birth weight groups [24]. Decreasing PatO_2_ at altitude is also associated with an increased prevalence of pre-eclampsia, a syndrome of maternal hypertension and proteinuria, which can progress to life-threatening seizures, as well as IUGR [25]. Chronic lung disease follows an accelerated course in hypoxic environments, resulting in a shorter interval between onset and death, and further increasing the incidence of right heart failure [26]. In short, lifetime exposure to low PatO_2_ exerts detrimental effects that may limit longevity, increase morbidity, and impair human reproduction. However, some populations have thrived at altitude and perhaps the survival of future generations of humans depends on the long-term adaptations observed in these people.

## Adaptation to hypoxia over generations

Populations that have occupied hypoxic environments for hundreds of generations appear to have undergone genetic adaptation leading to the expression of phenotypes that convey an enhanced ability to survive and reproduce under chronic hypoxic stress. Long-resident populations enjoy reduced incidence and severity of the high-altitude complications such as CMS and IUGR [20] compared to non-ancestral high altitude residents, and their superior physical performance at altitude is widely reported anecdotally and demonstrated by increased maximal oxygen consumption on exercise testing [27, 28].

Three populations have occupied highlands of 3500–4000 m above sea level for millennia. Current best estimates place Tibetan plateau settlement first (25,000 years ago) followed by that of the Andean Altiplano (12,000 years ago) with a range of values given for colonization of the Ethiopian plateau (between 5000 and 70,000 years ago), although we cannot prove genetic continuity between original colonizers and modern-day populations [29]. Specific physiological traits, related to the oxygen transport pathway or oxygen utilization, have been identified in these populations, but the composite of traits is different in each. For example, hemoglobin concentration is elevated in high-altitude Andeans, but remains close to typical lowlander values at sea level in Tibetan and Ethiopian highlanders up to altitudes of 4000 m, with only minimal increment upon further ascent [30]. On the other hand, SpO_2_ is lower in high-altitude Tibetans and Andeans compared to Ethiopians [30]. Tibetan and Andean highlanders have been the most extensively studied, with comparatively limited information available about their Ethiopian counterparts, and the main phenotypic differences between the three populations are summarized in Table 1. There is a lack of consensus regarding some of these differences, and we have much to learn about how and to what extent each phenotype actually contributes to improved function or survival in hypoxic conditions.

**Table 1:** Physiological responses to sustained exposure to hypobaric hypoxia in different native populations (in comparison to values seen at sea level) [29, 46, 47]. Adapted from [33]

Phenotype	Andean	Tibetan	Ethiopian
Resting ventilation	No increase	50% higher	Not reported
Hypoxic ventilatory response	Blunted (low)	Similar to sea level (high)	Not reported
Arterial oxygen saturation	Elevated	No increase	Elevated
Hemoglobin concentration	Elevated	No increase (up to 4000 m)	No increase (up to 4000 m)
Pulmonary arterial pressure	Elevated	Minimal increase	Elevated
Nitric oxide levels	Elevated	Markedly elevated	Not reported
Birth weight	Elevated	Elevated	Not reported
CMS incidence	Frequent	Rare	Very rare

Many approaches have been applied to uncover the genetic basis of these hypoxia-adapted phenotypes. High-altitude populations appear to have undergone positive selection in many genes that are involved in the HIF signaling cascade, which co-ordinates the cellular and systemic response to hypoxia. Examples of such genes are summarized in Fig. 4. In most instances, the precise function these genetic variants is yet to be revealed, but in some instances, putative mechanisms are beginning to emerge. For example, a variant of the EPAS1 gene (which encodes the alpha subunit of the HIF-2 transcription factor) has been demonstrated at increased frequency in high-altitude Tibetans. The selected variant actually down-regulates HIF targets, including erythropoietin, and is associated with lower hemoglobin concentrations [31]. It has thus been proposed that it may promote survival in hypoxic conditions by protecting against CMS and improving microcirculatory flow and local oxygen delivery due to reduced blood viscosity. The Tibetans inherited this gene from an ancient human race called the Denisovans, prior to their extinction 40,000 years ago [32]. It has been identified in only one other population on Earth, the Han Chinese, from which the Tibetans split less than 3000 years ago. In this time, the frequency of the gene in the two populations has diverged significantly: it is present in only 9% of Han but in 87% of Tibetans, the fastest known example of Darwinian evolution of humans [32]. The timeframe over which this significant genetic population change occurred is roughly equivalent to the time over which the model predicts global PatO_2_ to halve, and offers some insight into how quickly humankind might be able to adapt to the oncoming hypoxic selection pressure.

Any predictions about the nature of the human race in an oxygen-deplete future using the genetics of present-day high altitude populations is hampered by the fact that different genes appear to have undergone positive selection in each, with no overlap in the variants expressed by the Tibetans, Andeans, or Ethiopian highlanders [33]. Genomic analysis of Andean populations has revealed at least 40 candidate genes involved in the HIF pathway or hypoxia-related genes, including PRKAA1 (which codes for a subunit of adenosine monophosphate-activated protein kinase, and may influence fetal growth) [33, 34]. Natural selection in many genes involved in the same pathways has been demonstrated in high-altitude Tibetans, but the specific genes are at different loci or constitute different variants, such as EGLN1 (which encodes prolyl hydroxylase 2, the oxygen-dependent modulator of the HIF alpha subunits [35]). Ethiopian highlanders show positive selection in different genes again, this time including BHLHE41, which may be both a target and a modifier of HIF-1 alpha [33, 36]. One possibility is that different populations have followed different paths towards hypoxic adaptation, influenced by other environmental variables in each location (such as temperature or food availability), and population factors such as the genetic variation in the original settlers (contributing to genetic drift) and access to other gene pools (contributing to genetic flow). A second explanation is that they represent different time points on the same journey towards an optimally adapted phenotype, with duration and degree of hypoxic exposure different in each region. If we accept the second explanation, then the Tibetans, exposed to the greatest degree of hypoxic stress for the longest time, would represent the current pinnacle of long-term hypoxic adaptation. This is corroborated by the fact that Tibetans have a lower incidence of CMS and IUGR than their shorter-resident Andean counterparts [20]. The nature and rate of human adaptation to future atmospheric hypoxia will depend on stochastic events and making predictions is dogged by uncertainty, but the rate of oxygen decline that is projected by the parabolic model (PatO_2_ falling by 50% over the next 3500 years) may not provide sufficient time for the development of a Tibetan phenotype, but perhaps just enough to allow an Andean pattern of traits to emerge.

## Hypoxia survival limits and human extinction

Even with genetic and phenotypic adaptation, the parabolic decline described by this mathematical model predicts a scenario in which atmospheric oxygen concentration falls to levels below the threshold where human survival and reproduction may be sustained. Defining this point in terms of oxygen concentration is difficult, and our hypothesis is based on the highest elevations known to sustain lifelong human habitation. The highest permanent settlement in the present day is the Peruvian village of La Riconada, at an altitude of 5100 m, which has around 30,000 inhabitants [37]. Native villagers have survived there for at least 40 years and current residents have successfully gone through child birth to create the next generation at this altitude [38], however, it is not known whether the birth rate can sustain this population indefinitely. The highest permanent settlement on record is the (now abandoned) Chilean mining village of Quilcha (5340 m), which was discovered by the 1935 International High Altitude Expedition to Chile [39]. It has been argued that this represents the upper limit of long-term human habitation, because the residents chose to sleep at this elevation and make a daily ascent to the mine above. PatO_2_ at the Quilcha settlement is 11.3 kPa (slightly higher than 50% of the current PatO_2_ at sea level). The parabolic deoxygenation model described here predicts that PatO_2_ at sea level will reach this threshold in ~3600 years from now. During this time, the human species is likely to undergo further positive selection for physiological phenotypes conveying survival advantage in hypoxic conditions. Studies of high-altitude residents tell us that while such adaptations may enable us to function relatively well in an atmosphere that contains just over half the oxygen we breathe today; many will suffer the long-term consequences. Higher rates of maternal pre-eclampsia and death, increased perinatal mortality, low birth-weights (and the myriad consequences of this in adulthood) and escalating pulmonary disease will curtail life expectancy and population growth. Those individuals with independent comorbidities, particularly chronic respiratory and cardiac disease, may suffer exacerbation of their symptoms, reduced function, and reduced length of life. Highlanders may be forced to descend as life becomes intolerable at hypobaric elevations, therefore reducing the surface of Earth that we can populate. The burden of ill health will begin to overwhelm the capability of healthcare services. The last prevailing human phenotypes may resemble those of current high-altitude populations: with enhanced abilities to extract precious oxygen from the atmosphere or deliver it to the tissues, and perhaps superior cellular mechanisms to improve efficiency of oxygen use and defend against hypoxic stress.

It is important to stress that the parabolic model described here is mathematical rather than geophysical [5]. Other authors have disputed the idea that global deoxygenation on a catastrophic scale is possible [40]. One of the key reasons cited for this is that the determining factor in global oxygen decline is fossil fuel usage and current estimates predict that oil, coal, and gas stocks will last 35, 107, and 37 years, respectively [41]. Thus, is it plausible that the increased fossil fuel usage in recent years has caused a temporary acceleration of the deoxygenation phenomenon, which will resolve once reserves have been exhausted. This scenario would predict a very different decline in atmospheric oxygen from the one we have described, with a fall of only a fraction of a percent in 4400 years [42]. Consensus in this area has not yet been achieved, but the need to understand the limits of long-term human survival under progressively hypoxic conditions cannot be questioned, whether we are considering the persistence of the human race in the mathematical model discussed here, or other contexts in which atmospheric oxygen may become scarce, such as future long-term space expeditions. Other environmental changes may also impact the ability of humans to acclimatize to hypoxia during global deoxygenation and these include the rise in both temperature and concentration of carbon dioxide. It is hard to predict the precise effect of these additional physiological stressors but both are likely to reduce further our chances of long-term survival. In particular, rising carbon dioxide levels could lead to metabolic problems if individuals fail to adapt to this adequately. High blood carbon dioxide levels (hypercarbia) can cause acidosis, hypertension, and tachycardia. Those with underlying lung chronic disease may suffer greatest from this. In a simple, short-term experimental model of Earth’s atmosphere, a novel experiment that used plants to generate oxygen and consume carbon dioxide in a sealed hypoxic chamber noted a high carbon concentration (0.66%) towards the end of the 48-h experiment [43].

The atmospheric changes will also impact other animals on Earth, and failure to adapt will result in extinction both on land and in the seas and oceans. All aerobic life forms will suffer as oxygen is removed from the atmosphere. In addition, plant metabolism may also be detrimentally affected. Already, the rising carbon dioxide concentration has been predicted to reduce the rate of photorespiration, and a falling oxygen concentration may exacerbate this. While the overall effect of a reduction in photorespiration remains unclear, we do know that complete removal of this pathway could lead to metabolic disaster for the plants that use it [44].

## Conclusions

Progressive asphyxiation of the planet would ultimately lead to the demise of humankind through escalating infant mortality and eventually complete failure to reproduce. Perhaps technological advancement could permit the continuation of life within biospheres in the short term [45], but beyond this, the outside world would become a barren and inhospitable place. It is not possible to predict with certainty the threshold value at which mass extinction becomes inevitable, but we have no evidence that humans can persist for more than a generation in an atmosphere containing half the amount of oxygen currently available at sea level, a situation that, according to a new model, could be upon us in a few thousand years. Unless the process of global deoxygenation is reversed, either by increasing oxygen production or by reducing its consumption, the human race, as obligate aerobes, will be left behind forever, our domination of this planet a brief footnote in its history.
